# Supplementary material for: AI‐Powered Multimodal Modeling of Personalized Hemodynamics in Aortic Stenosis
Source: Adv Sci (Weinh). 2024 Dec 12;12(5):2404755. doi: 10.1002/advs.202404755 (PMC11791996; doi:10.1002/advs.202404755)
Supplement: Supplementary file 1 — Supporting Information [file ADVS-12-2404755-s003.pdf]

ADVANCED SCIENCE
Open Access

Supporting Information

for *Adv. Sci.*, DOI 10.1002/advs.202404755

AI-Powered Multimodal Modeling of Personalized Hemodynamics in Aortic Stenosis

Caglar Ozturk, Daniel H. Pak, Luca Rosalia, Debkalpa Goswami, Mary E. Robakowski, Raymond McKay, Christopher T. Nguyen, James S. Duncan and Ellen T. Roche**

Supporting Information

AI-Powered Multimodal Modeling of Personalized Hemodynamics in Aortic Stenosis

Caglar Ozturk[†], *Daniel H. Pak*[†], *Luca Rosalia*, *Debkalpa Goswami*, *Mary E. Robakowski*, *Raymond McKay*, *Christopher T. Nguyen*, *James S. Duncan**, and *Ellen T. Roche**

[†] *These authors contributed equally to this work.*

Supplementary Figures

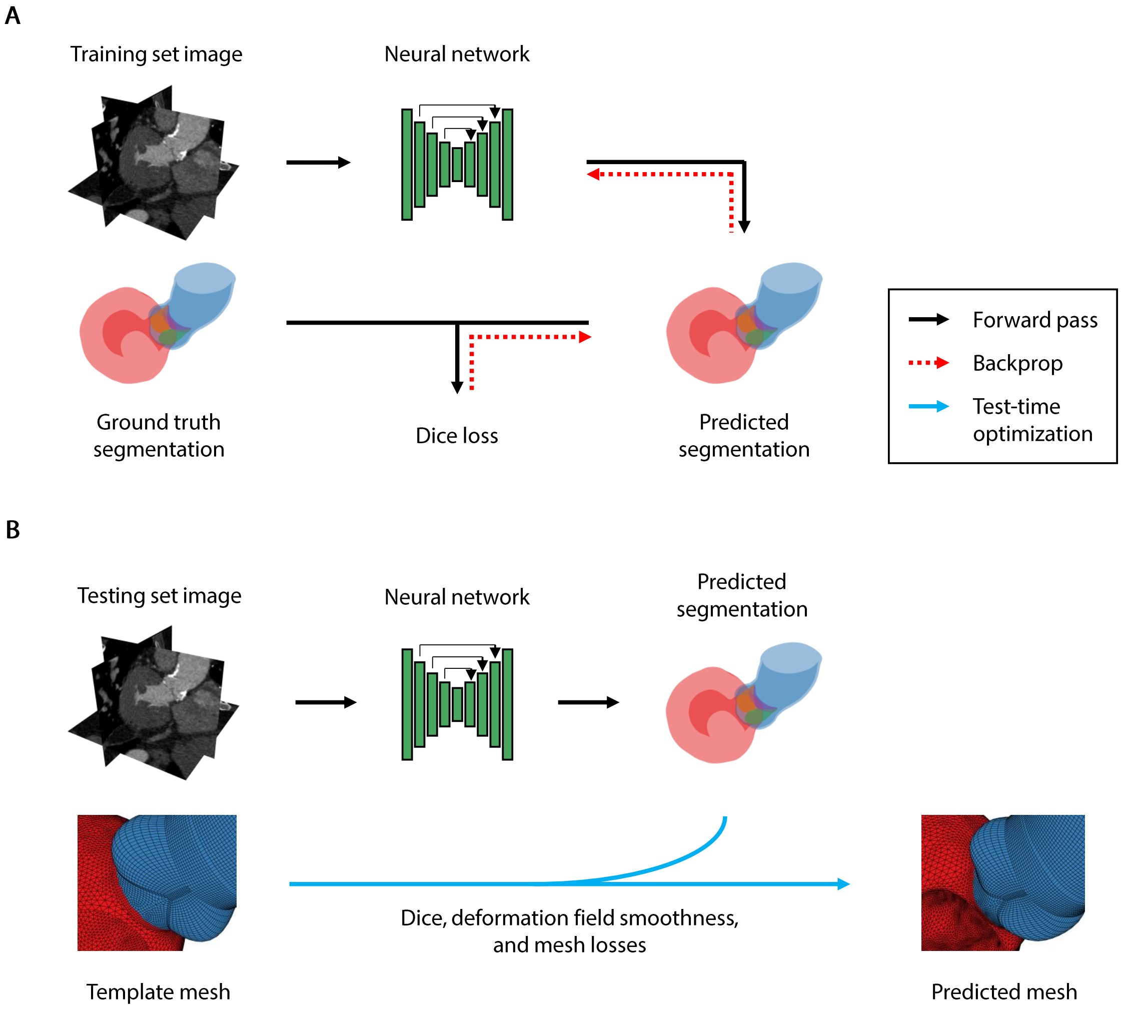

Figure S1. Flowchart of the conventional registration algorithm. (A) Training and (B) testing phases.

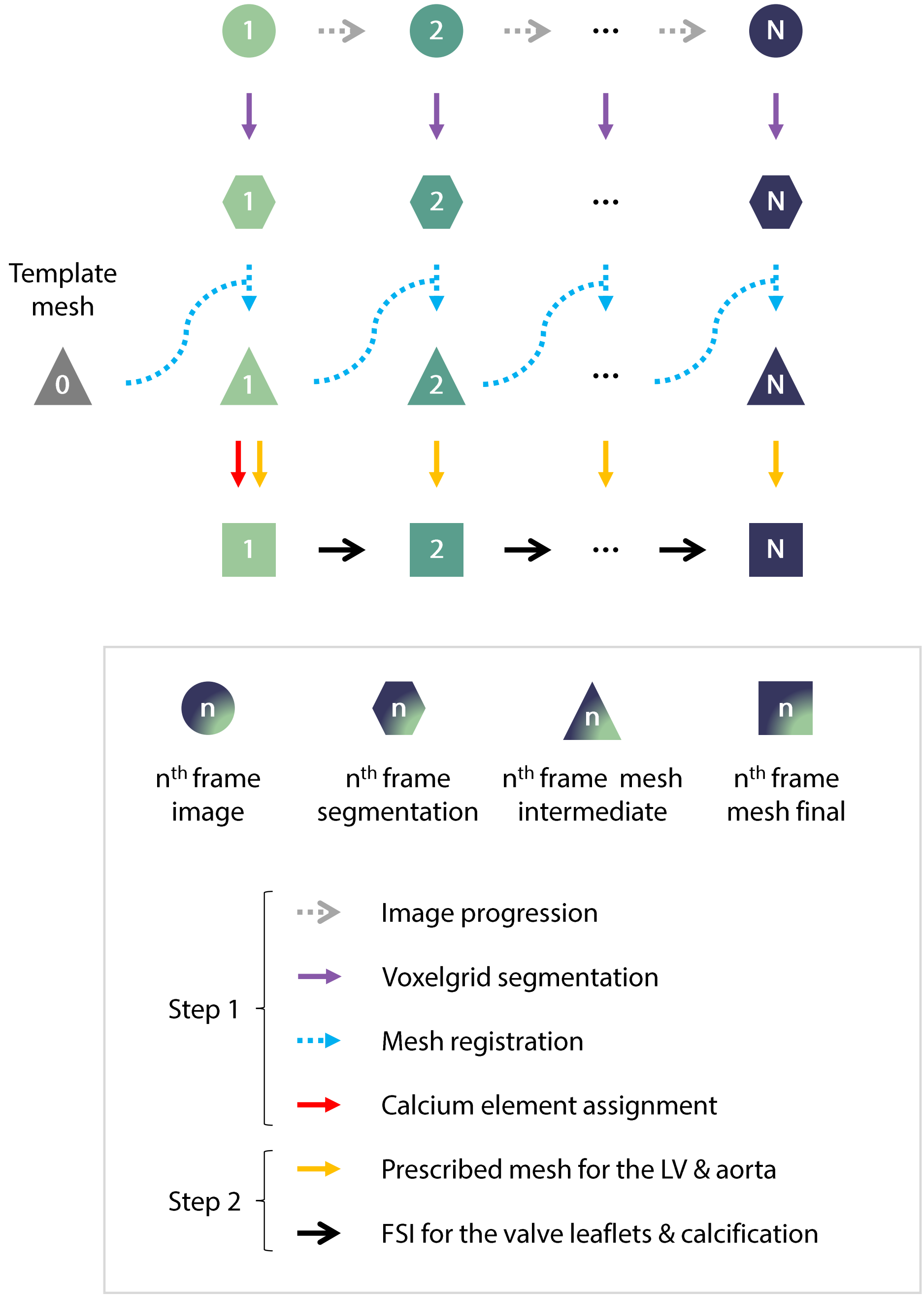

Figure S2. Application of the conventional registration algorithm for dynamic mesh generation.

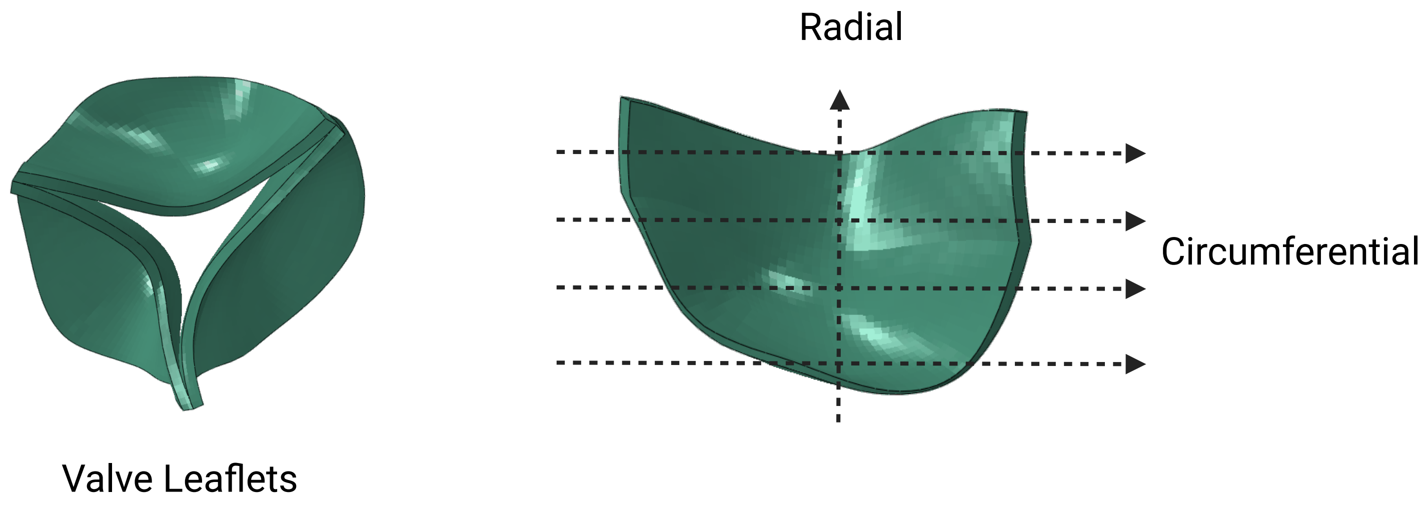

Figure S3. Anisotropic hyperelastic material definition of aortic valve (AV) leaflets and fiber orientation assignment.

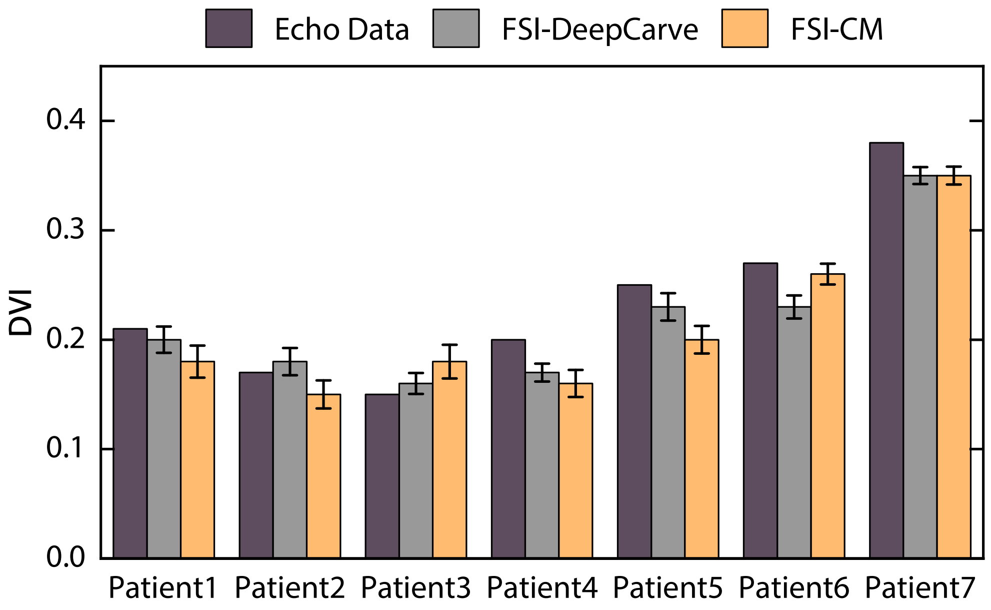

Figure S4. Evaluation of dimensionless velocity index (DVI) as the ratio of the left ventricular outflow tract (LVOT) velocity and the maximum velocity across the valve.

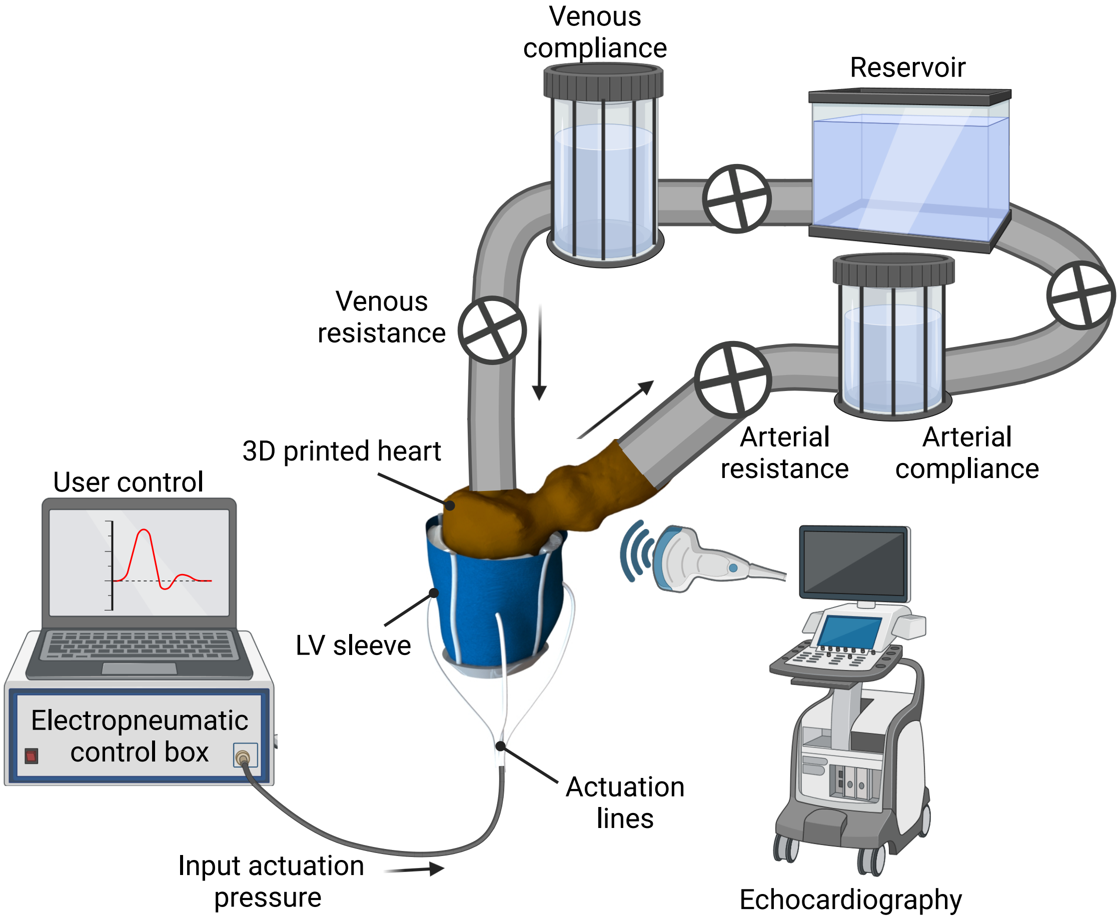

Figure S5. Schematic of mock circulatory flow loop.

List of Supplementary Videos

Movie S1. Image sequence analysis with DeepCarve algorithm on dynamic CT data.
Movie S2. Implementation of the conventional registration method.
Movie S3. Implementation of the 3D printing mesh conversion algorithms.
Movie S4. In-silico hemodynamic assessment of patient 1 using DeepCarve-FSI framework.
Movie S5. Echocardiographic imaging of the left ventricle and continuous wave Doppler imaging of the velocity waveforms across the aortic valve during soft robotic left ventricular sleeve actuation.
